# 

**Published:** 1874-06

**Authors:** 


### Table of Contents.

### ORIGINAL COMMUNICATIONS.

INTESTINAL OBSTRUCTIONS: A Safe and Ready Method—*By Robert Battey, M. D., Atlanta, Ga.* ........ 129
HEMORRHAGIC, OR MALIGNANT BILIOUS FEVER—*By W. F. DeWitt, M.D., Atlanta, Ga.* ........ 142

### MEDICAL SOCIETIES.

ATLANTA ACADEMY OF MEDICINE—Vaginal Ovariotomy—Ovariotomy—Ovarian Adhesions—Large Fatty Tumor—Abortion—Double Monster (photographic illustration)—Fracture of Patella, with Bony Union—Removal of Hemorrhoids ........ 145
ASSOCIATION MEDICAL OFFICERS of late Confederate Army and Navy.... 182

### OUR EXCHANGES.

The Brain Power of Man ........ 158
Report on Ovaries removed by Dr. T. G. Thomas ........
Faculty of Medicine in Geneva ........ 160
Nervous Mimicry of Organic Diseases ........ 161
Surgical Treatment of Aneurism ........ 163
Consultations in the Country ........ 164
Urethrotomy by Dr. F. N. Otis ........ 166
Paracentesis Thoracis—Tracheotomy, by Dr. Sayre ........
Morphia Strength of Laudanum ........ 167
Personal Atmosphere of the Physician ........ 168
Phytolacca Decandra in Mastitis ........ 169
Induction of Labor in Uræmia. ........ 170
Artificial Butter—Legal Responsibility of Consulting Physicians ........ 171
Ulceration of the Uterus, Dr. E. Kennedy—"Catching Cold" ........ 173
Statistics of Rheumatism, ........ 175
Vesico-Vaginal Fistula, No after Treatment ........ 176
Dr. O'Flanagan and his Wonderful Cures (poetical) ........ 178

### EDITORIAL.

Is Alcohol Food? ........ 190
We surrender Space—Alabama State Medical Association ........
Virginia Medical Monthly ........ 192

